# The Efficacy of Vaccination

**Published:** 1892-10-01

**Authors:** 


### THE EFFICACY OF VACCINATION.

Some time ago the surgeon of a small-pox hospital was asked to give his observations of the result of vaccinated patients whom he had treated. He found, during his twenty-five years' experience of 6,000 cases of post vaccinal small-pox that the percentage of deaths was (1) $21\frac{3}{4}$ in the case of persons vaccinated, but having no vaccine cicatrix; (2) $7\frac{1}{2}$ per cent. having one cicatrix; (3) $4\frac{1}{8}$ having two cicatrices; (4) $1\frac{3}{4}$ having three vaccine cicatrices; and (5) having four or more cicatrices, $\frac{3}{4}$ per cent.; whilst unvaccinated amounted to $35\frac{1}{2}$ per cent. There seems to be a growing objection to vaccination in some quarters; it would be well if actual statistics on this point were more fully known.

